# Rethinking Dengue Preparedness in the Era of Climate Change, Urbanisation, and Digital Health: A Structured Narrative Review

**DOI:** 10.3390/medicina62071333

**Published:** 2026-07-10

**Authors:** Marco Dettori, Giovanna Deiana, Alessandra Palmieri, Antonella Arghittu, Paolo Castiglia, Andrea Piana, Guglielmo Campus

**Affiliations:** 1 Department of Medicine, Surgery and Pharmacy, University of Sassari, 07100 Sassari, Italy; 2 Medical Management, Hygiene, Epidemiology and Hospital Infection, University Hospital of Sassari, 07100 Sassari, Italy; 3 Department of Cariology, Institute of Odontology, Sahlgrenska Academy, University of Gothenburg, 413 90 Gothenburg, Sweden; 4 Department of Cariology, Saveetha Dental College and Hospitals, Velappanchavadi, Chennai 600 077, India; 5 Department of Oral and Maxillofacial Sciences, Sapienza University of Rome, 00161 Rome, Italy

**Keywords:** dengue, public health, climate change, urbanisation, surveillance, digital health, artificial intelligence, mathematical modelling

## Abstract

*Background and Objectives:* Dengue is emerging as a multifaceted public health challenge that extends beyond traditional vector-borne disease frameworks. Climate change, rapid urbanisation, environmental transformation, global mobility, and digital ecosystems are progressively reshaping transmission dynamics, outbreak patterns, and preparedness needs worldwide. This narrative review aimed to examine dengue from an integrated public health perspective, focusing on climate-sensitive transmission, urban health, surveillance and preparedness, digital epidemiology, artificial intelligence (AI), and health communication. *Materials and Methods:* A structured narrative review was conducted through targeted literature searches in PubMed, Scopus, and Web of Science between April and May 2026. To this end, a series of separate thematic search strategies were developed to explore the principal conceptual domains addressed in the review. The synthesis was organised around five interconnected preparedness domains: climate change and environmental transformation; urbanisation and urban health; surveillance, vaccination, and integrated preparedness; digital health, artificial intelligence, and mathematical modelling; and health communication and community engagement. The retrieved literature was analysed using a thematic narrative synthesis approach. *Results:* The retrieved evidence indicated the progressive expansion and redefinition of dengue risk across both endemic and historically non-endemic regions. Climate variability, environmental transformation, rapid urbanisation, and increasing human mobility have emerged as interconnected drivers capable of influencing vector ecology, transmission dynamics, outbreak frequency, and healthcare system vulnerability. Urbanisation has been frequently associated with infrastructural inequalities, environmental degradation, inadequate water and waste management, and territorial conditions favourable to vector proliferation. The extant literature has also placed significant emphasis on the growing importance of integrated surveillance systems and early warning approaches combining epidemiological, environmental, climatic, entomological, and mobility-related data. Digital epidemiology, AI-based predictive models, and digital surveillance tools may contribute to strengthening outbreak forecasting and preparedness capacity, although important limitations related to data quality, interoperability, interpretability, and implementation remain. In parallel, misinformation, risk communication challenges, and digital communication ecosystems emerged as relevant factors influencing public perception, preventive behaviours, institutional trust, and adherence to public health interventions. *Conclusions:* Dengue is a systems-level public health challenge shaped by climate change, urbanisation, environmental disruption, human mobility, health-system preparedness, and digital ecosystems. Conventional vector-control strategies alone are unlikely to adequately address this growing complexity. Strengthening dengue preparedness should therefore be considered a broader indicator of public health resilience and long-term health-system adaptation.

## 1. Introduction

Dengue has emerged as one of the most rapidly expanding mosquito-borne viral diseases worldwide, posing an increasing challenge to public health systems in endemic regions and in areas previously considered non-endemic [1,2]. According to the World Health Organisation (WHO), the global incidence of dengue has risen dramatically over recent decades, accompanied by a progressive geographic expansion into tropical, subtropical, and even temperate areas [1]. Concurrently, the escalating frequency and severity of outbreaks have raised increasing concerns regarding healthcare preparedness, surveillance capacity, environmental management, and the resilience of public health systems [3].

Traditionally, dengue has been conceptualised predominantly as a mosquito-borne infectious disease largely influenced by the ecology and distribution of *Aedes* vectors. However, the contemporary epidemiological landscape is considerably more complex. *Aedes aegypti* is more strongly associated with urban domestic transmission, while *Aedes albopictus* is important for expansion into temperate areas, including Europe. The dynamic interplay of climate change, rapid urbanisation, demographic shifts, increasing human mobility, environmental transformation, and global interconnectedness is progressively reshaping patterns of dengue transmission and exposure [4,5,6]. In this context, a vector-centred interpretation alone may be insufficient to account for the interaction among environmental, urban, social, and healthcare-system determinants of dengue risk [4,5,6,7,8,9].

Urbanisation is a critical factor in this evolving scenario. The proliferation of urban growth, the presence of informal settlements, inadequate water supply and waste management, high population density, and environmental degradation can create favourable ecological conditions for vector proliferation and facilitate transmission in densely populated urban settings [5,7]. At the same time, contemporary urban environments are characterised by structural tensions between economic development, environmental sustainability, and public health protection. Urban sprawl and uncontrolled territorial expansion may further contribute to environmental modifications capable of influencing vector ecology, human exposure patterns, and inequalities in access to preventive measures and healthcare services [8,9].

In parallel, increasing attention has been directed toward the potential role of digital health technologies, artificial intelligence (AI), and data-driven surveillance systems in supporting dengue preparedness and outbreak response [10,11]. Digital health refers broadly to the use of digital technologies, platforms, and data systems to support public health practice, healthcare delivery, surveillance, communication, and decision-making. Digital epidemiology refers more specifically to the use of digital data sources and computational methods to monitor, analyse, and anticipate population-level disease patterns, including data derived from online searches, social media, mobility systems, remote sensing, and real-time surveillance platforms [10,11,12].

The growing availability of environmental, climatic, epidemiological, entomological, and mobility-related data has enabled the development of predictive models intended to enhance early warning, monitor transmission trends, and support evidence-informed public health decisions [12]. Digital communication ecosystems comprise the interconnected platforms, institutions, media actors, health authorities, and users through which health information is produced, circulated, interpreted, and contested. These ecosystems influence public perception, health literacy, risk communication, preventive behaviour, and the dissemination of both accurate information and misinformation during infectious disease outbreaks [13].

Despite the expanding literature on dengue epidemiology and vector control, many contributions remain predominantly focused on entomological, virological, or outbreak-specific aspects [14,15,16,17]. Climate change, urbanisation, surveillance, vaccination, digital technologies, and health communication are also frequently examined as separate domains. Fewer studies have considered how these dimensions interact within a single preparedness framework and how their convergence affects healthcare systems, public health governance, and response capacity across endemic and emerging-risk settings. The added value of the present review therefore lies not in treating each determinant independently, but in integrating them as interconnected components of dengue preparedness and health-system resilience.

Within this framework, a One Health perspective is relevant. Dengue risk emerges from interactions among human populations, mosquito vectors, climatic conditions, urban environments, mobility, and public health systems. Although dengue is not a zoonosis in its predominant urban transmission cycle, One Health provides an appropriate operational framework for integrating environmental monitoring, vector surveillance, human epidemiology, community participation, and intersectoral preparedness.

From this perspective, the present review examines dengue as a systems-level public health challenge shaped by interacting environmental, urban, epidemiological, technological, communicative, and healthcare-system factors. Its objective is to integrate these domains within a preparedness-oriented framework and to identify their operational implications for surveillance, prevention, vaccination, risk communication, community engagement, and healthcare-system adaptation. Particular attention is given to the transition from reactive outbreak control toward anticipatory and coordinated preparedness.

## 2. Materials and Methods

### 2.1. Study Design

Given its interpretive and integrative objective, this study was designed as a structured narrative review rather than a systematic review. The review followed a predefined thematic framework and explicit eligibility criteria. No formal risk-of-bias assessment or quantitative evidence synthesis was performed, as the objective was to integrate heterogeneous evidence across multiple public health domains rather than estimate pooled effects.

The selected literature was analysed using a thematic narrative synthesis approach. The primary objective of the synthesis was to provide an integrated public health interpretation of dengue within contemporary environmental, urban, and digital contexts, rather than to quantitatively synthesise the available evidence.

### 2.2. Literature Search Strategy

The literature search was performed using PubMed, Scopus, and Web of Science. Searches were conducted between April and May 2026 and were updated on 13 May 2026. They focused on studies investigating dengue in relation to five predefined thematic domains.

The search strategy was developed to capture interdisciplinary contributions spanning epidemiology, environmental health, urban health, digital epidemiology, mathematical modelling, healthcare preparedness, and health communication. Separate thematic search blocks addressed: (i) climate change and environmental transformation; (ii) urbanisation and urban health; (iii) surveillance, vaccination, and integrated preparedness; (iv) digital health, artificial intelligence, and mathematical modelling; and (v) health communication and community engagement. Each block combined “dengue” with domain-specific terms using Boolean operators. The main thematic search strategies used across the three databases are reported in Supplementary Table S1. The strategy was informed by established approaches to structured narrative reviews and adapted to the interdisciplinary scope of the present study [18,19].

### 2.3. Eligibility Criteria

Eligible records included original epidemiological and public health studies, narrative and systematic reviews, modelling studies, and articles addressing climate-sensitive transmission; urban and environmental determinants of dengue; surveillance, vaccination, and preparedness; digital epidemiology and artificial intelligence; mathematical modelling; health communication; community engagement; or misinformation. Only English-language publications were considered. Studies focused exclusively on laboratory research, vector biology, entomology, or highly technical molecular analyses without direct public health relevance were excluded, as were studies not aligned with the thematic objectives of the review.

Publications issued between January 2021 and May 2026 were considered recent. Earlier studies were retained when they represented landmark contributions, established methodological approaches, or provided essential historical context for the interpretation of current dengue preparedness.

### 2.4. Study Selection and Data Organisation

**Figure 1:**
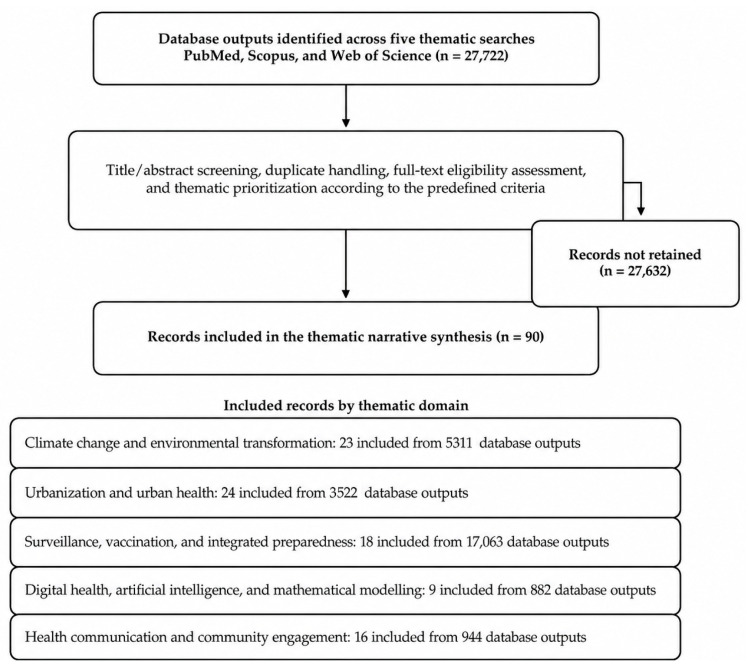
Thematic study-selection flow.

Records retrieved from PubMed, Scopus, and Web of Science were exported and organised according to the five predefined thematic domains. Duplicate records were handled within each thematic search block. Titles and abstracts were screened independently by two reviewers (G.D. and A.Pal.), and potentially relevant records were subsequently assessed at the full-text level. Disagreements concerning eligibility or thematic attribution were resolved through discussion and, when necessary, consultation with a third author (M.D.). Full texts were retained on the basis of direct relevance to the predefined domains, methodological robustness, contribution to the public health interpretation of dengue, and the need to include both recent empirical evidence and landmark studies. Reasons for exclusion at the full-text stage included insufficient relevance to the review objectives, an exclusive focus on laboratory or molecular mechanisms without direct public health implications, duplication of evidence, and absence of retrievable full text. The study-selection process is presented in Figure 1.

The figure summarises the cumulative database outputs retrieved across the five predefined thematic searches, the screening and eligibility process, and the records retained for the narrative synthesis. Because searches were conducted separately across databases and thematic domains, overlap among database outputs may occur; therefore, the reported identification counts should not be interpreted as numbers of unique records.

Overall, the targeted searches retrieved a large and heterogeneous body of literature spanning infectious diseases, epidemiology, environmental health, urban health, preparedness, digital epidemiology, AI, and health communication. The raw database outputs were used to identify a smaller set of representative records selected for thematic synthesis within each domain (Table 1).

## 3. Results and Discussion

The thematic synthesis was organised around five closely interconnected dimensions of dengue preparedness: (i) climate change and environmental transformation; (ii) urbanisation and urban health; (iii) surveillance, vaccination, and integrated preparedness; (iv) digital health, artificial intelligence, and mathematical modelling; and (v) health communication and community engagement. Taken together, the reviewed studies suggest that dengue risk is shaped by interactions among environmental suitability, urban infrastructure, mobility, population vulnerability, surveillance capacity, public trust, and institutional response.

### 3.1. Climate Change and Environmental Transformation

The reviewed evidence indicates that the ecological and geographical boundaries historically associated with dengue transmission are becoming unstable [20,21,22,23,24,25,26]. Although dengue remains concentrated in tropical and subtropical regions, changes in temperature, rainfall, humidity, land use, urbanisation, and human mobility are modifying vector suitability and the seasonal conditions under which transmission may occur. This does not imply that climatic suitability alone determines transmission; rather, it indicates that environmental change is interacting with vector ecology, population immunity, mobility, infrastructure, and surveillance capacity to alter the distribution and predictability of risk.

Climate suitability should not be directly equated with transmission, as dengue incidence is influenced by nonlinear interactions among vector ecology, viral factors, population immunity, human mobility, urban infrastructure, and surveillance capacity. The retrieved studies collectively suggest the emergence of complex ecological scenarios characterised by altered rainfall patterns, prolonged heatwaves, humidity changes, extreme climatic events, and progressive modifications in vector suitability across diverse geographical settings [27,28,29,30,31,32,33,34,35]. The aforementioned environmental pressures may exert a simultaneous influence on mosquito survival, vector density, viral replication, and transmission efficiency. Consequently, these pressures may generate unstable and difficult-to-predict epidemiological conditions.

Several high-impact studies identified in the present review further emphasise that the relationship between climate change and dengue cannot be interpreted solely through an entomological perspective [36,37,38]. The expansion of transmission risk appears deeply interconnected with urban growth, demographic pressures, international mobility, environmental degradation, and heterogeneous preparedness capacities across healthcare systems. In this context, climate change functions less as an isolated determinant than as an amplifying driver interacting with pre-existing environmental and public health vulnerabilities.

Another recurrent theme concerns the growing uncertainty surrounding future transmission dynamics. Climate-sensitive redistribution models suggest that regions historically considered climatically unsuitable for dengue transmission may progressively become vulnerable to seasonal or localised outbreaks [39,40,41,42]. These findings raise important implications for surveillance systems and preparedness strategies, particularly in areas where healthcare infrastructures, vector monitoring systems, and public awareness remain historically oriented toward other infectious disease priorities.

Species-distribution and ecological-niche models, including maximum entropy (MaxEnt) approaches, have also been used to estimate the potential distribution of *Aedes aegypti* and *Aedes albopictus* under present and future climatic conditions. These models can help identify areas in which temperature, rainfall, humidity, and land-cover conditions may become suitable for vector establishment. However, their projections depend strongly on the quality and representativeness of occurrence data, the selection of environmental variables, spatial resolution, and assumptions regarding future climate scenarios. They should therefore be interpreted as estimates of potential environmental suitability rather than direct predictions of dengue transmission [38,41].

The literature also reflects a gradual conceptual transition from reactive outbreak management toward anticipatory and integrated surveillance approaches. Several studies highlight the importance of combining climatic, epidemiological, environmental, and entomological information within early warning frameworks capable of supporting timely preparedness measures and more efficient resource allocation [39,40]. In this evolving scenario, climate-sensitive surveillance is emerging as a strategic component of infectious disease prevention and control rather than merely an environmental monitoring activity.

Of particular relevance is the growing attention devoted to Mediterranean and temperate regions. Recent experiences in Southern Europe have reinforced concerns regarding the interaction among climate variability, vector adaptation, imported cases, and local surveillance capacity [21,27,31,32]. Cyprus provides a particularly important example because the recent detection and introduction of both *Aedes aegypti* and *Aedes albopictus* illustrates how vector ecology in the Mediterranean is changing in ways that may increase the potential for local transmission [43]. Recent updates from the European Centre for Disease Prevention and Control (ECDC) further indicate that the distribution of invasive *Aedes* mosquitoes in Europe is rapidly evolving, reinforcing the need for continuous entomological surveillance in Mediterranean and other climatically receptive areas [44]. The presence of competent vectors does not in itself establish endemic transmission, but it increases the importance of coordinated entomological surveillance, rapid diagnosis of imported cases, cross-border information sharing, and preparedness for localised outbreaks. These developments show that Mediterranean preparedness can no longer rely on the assumption that dengue risk remains confined to historically endemic regions.

The strength of this evidence is nevertheless heterogeneous. Many studies rely on ecological associations, retrospective analyses, or model-based projections, and their findings are sensitive to spatial resolution, variable selection, climate scenarios, surveillance quality, and assumptions regarding vector competence and human behaviour [27,28,29,30,31,32,33,34,35,36,37,38,39,40,41,42]. Vector presence, population immunity, imported cases, diagnostic capacity, and public health response remain decisive modifiers of actual transmission risk. Accordingly, confidence is greatest for the general association between climatic conditions and vector suitability, whereas estimates of future geographic expansion and outbreak magnitude remain more dependent on modelling assumptions, local surveillance quality, and contextual conditions.

Overall, the retrieved evidence consistently supports the interpretation of dengue as a climate-sensitive public health challenge whose future evolution will likely depend on the interaction among environmental transformation, urbanisation processes, surveillance capacity, preparedness strategies, and the adaptive resilience of healthcare systems.

### 3.2. Urbanisation and Urban Health

Urbanisation emerged not simply as a demographic phenomenon, but as one of the major structural determinants shaping contemporary dengue transmission dynamics [45,46,47,48,49,50,51]. Several studies frame dengue as a disease strongly influenced by the interaction among rapid urban growth, environmental pressure, infrastructure vulnerability, and population density, particularly in large metropolitan and peri-urban settings.

A recurrent pattern identified across the literature concerns the role of unplanned urban expansion and heterogeneous urban development in facilitating ecological conditions favourable to vector proliferation. Informal settlements, intermittent water supply, uncovered domestic water-storage containers, discarded receptacles, insufficient waste management, overcrowding, and limited sanitation infrastructure may collectively increase the availability of mosquito breeding sites and amplify transmission risk [52,53,54,55,56]. *Aedes aegypti* commonly exploits artificial containers containing relatively clean or lightly contaminated water in and around households, although breeding-site use varies according to local environmental and infrastructural conditions. In many rapidly expanding urban areas, these conditions coexist with profound social and spatial inequalities, generating heterogeneous exposure patterns even within the same metropolitan context.

Importantly, urbanisation should not be interpreted exclusively through population density metrics. Several contributions increasingly emphasise broader urban health and built environment perspectives that consider mobility patterns, land-use transformation, environmental fragmentation, access to healthcare services, and territorial organisation [57,58,59,60,61]. Within this framework, urban sprawl and uncontrolled territorial expansion may indirectly contribute to environmental and social conditions capable of influencing infectious disease vulnerability and preparedness capacity.

Another relevant aspect emerging from the selected studies concerns the growing convergence between urban health and climate-sensitive infectious disease research. High-density urban environments may amplify the effects of heat stress, water scarcity, environmental degradation, and extreme weather events, potentially interacting with vector ecology and transmission dynamics [62,63,64,65]. This interaction appears particularly critical in settings characterised by rapid urban transition and limited adaptive public health capacity.

Urban heat islands may further modify local mosquito ecology by producing temperatures that differ substantially from those recorded by regional meteorological stations. Warmer urban microclimates can influence larval development, adult survival, biting activity, the duration of the gonotrophic cycle, and the extrinsic incubation period of dengue virus. These effects are not uniformly favourable to mosquitoes, because temperatures exceeding species-specific thresholds may reduce survival and reproductive fitness. Urban heat should therefore be considered together with water availability, vegetation, housing characteristics, shading, and small-scale microclimatic variation when assessing intra-urban dengue risk [66,67].

At the same time, the literature highlights how urbanisation may also influence the effectiveness of surveillance and prevention strategies. Population mobility, commuting patterns, tourism, and global connectivity complicate traditional outbreak containment models, reinforcing the need for territorially adaptive and integrated preparedness approaches [68,69,70]. In this evolving scenario, dengue progressively emerges not only as a vector-borne disease, but also as an indicator of the broader relationship between urban transformation, environmental sustainability, and public health resilience.

The available evidence is also limited by substantial heterogeneity in the definition and measurement of urbanisation, informal settlements, built environment, population density, and infrastructural deprivation [45,46,47,48,49,50,51,52,53,54,55,56,57,58,59,60,61,62,63,64,65,66,67,68,69,70]. Many studies are cross-sectional or ecological and cannot establish causal pathways between specific urban characteristics and transmission. Future research should therefore combine fine-scale environmental, social, entomological, and mobility data to identify which urban conditions are most relevant for targeted prevention.

Overall, the available evidence supports the interpretation of urbanisation as a central system-level dimension in contemporary dengue epidemiology. Beyond vector ecology alone, the literature points toward the importance of understanding how urban environments, territorial organisation, and social inequalities may collectively shape infectious disease vulnerability, preparedness capacity, and long-term public health sustainability.

### 3.3. Surveillance, Vaccination, and Integrated Preparedness

Among all thematic domains explored in the present review, surveillance and preparedness generated the largest and most consolidated body of literature across all databases. This finding reflects the progressive recognition of dengue not only as a vector-borne infectious disease, but also as a major preparedness challenge for contemporary public health systems [2,11,71,72,73,74,75,76].

A recurrent theme concerns the limitations of reactive outbreak-oriented approaches. Several studies suggest that traditional surveillance systems based predominantly on retrospective case notification may no longer be sufficient in unstable epidemiological contexts characterised by climatic variability, rapid mobility patterns, and expanding vector suitability [72,73,74,75,76,77,78]. In response, the literature progressively shifts toward integrated and anticipatory surveillance models capable of combining epidemiological, environmental, climatic, entomological, and mobility-related information.

The available evidence also highlights the growing importance of early warning systems in supporting outbreak preparedness and resource allocation. Predictive surveillance frameworks integrating seasonal climatic forecasts, vector monitoring, and epidemiological indicators have shown increasing potential for anticipating transmission trends and supporting timely public health interventions [12,39,56,77,78,79,80]. However, several authors underline that predictive capacity alone is insufficient in the absence of adequate preparedness infrastructures, intersectoral coordination, and adaptive healthcare responses.

Within this broader preparedness framework, vaccination represents an additional preventive component that must be coordinated with surveillance, vector control, communication, and healthcare-system planning.

Dengue vaccination has become an important component of preparedness in endemic and high-burden settings. However, its effects on population-level transmission depend on vaccine characteristics, age-specific eligibility, baseline serostatus, vaccination coverage, circulating serotypes, duration of protection, and the epidemiological context in which programmes are implemented. The experience with Dengvaxia demonstrated the importance of carefully considering previous dengue exposure, safety, eligibility, and public communication, while the introduction of Qdenga has expanded the range of available preventive options. Vaccination should therefore be implemented as part of an integrated strategy that includes epidemiological and safety surveillance, transparent risk communication, clinical preparedness, community engagement, and sustained vector control. Vaccination may reduce disease burden and, under appropriate conditions, contribute to lower transmission, but it does not remove the need for entomological surveillance and environmental prevention [81,82].

The public health value of vaccination therefore depends on its integration with surveillance systems capable of monitoring serotype circulation, age-specific incidence, vaccine uptake, breakthrough infections, adverse events, and changes in disease severity. Vaccination policy also requires transparent communication because previous controversies have demonstrated how safety concerns, uncertainty, and inconsistent messaging can affect confidence not only in dengue vaccines but in immunisation programmes more broadly [81,83].

Another major issue concerns the marked heterogeneity of preparedness capacities across countries and regions. The literature consistently suggests that dengue outbreaks may disproportionately affect healthcare systems characterised by fragmented surveillance networks, delayed reporting mechanisms, insufficient vector monitoring programmes, and limited diagnostic capacity [71,73,84,85,86]. These vulnerabilities appear particularly relevant in rapidly urbanising settings and in regions undergoing environmental and climatic transitions capable of altering historical transmission patterns.

A One Health approach provides an operational framework for integrating human epidemiological surveillance with entomological, climatic, environmental, and territorial information. In the context of dengue, its value lies less in the management of animal reservoirs than in the coordination of public health authorities, environmental services, vector-control programmes, laboratories, urban planners, healthcare institutions, and communities. Such coordination may support earlier risk identification, more targeted vector control, improved environmental management, and more coherent preparedness across sectors [74,87,88,89]. Operationalization requires interoperable data-sharing arrangements, predefined alert thresholds, joint risk assessments, and protocols assigning responsibilities for signal verification, risk communication, vector control, and healthcare response. Regular multisectoral exercises and feedback mechanisms are also needed to ensure that integrated information leads to timely action rather than remaining confined to parallel surveillance activities.

At the same time, globalisation and increasing human mobility further complicate surveillance activities. Imported cases, tourism flows, migration, and international travel may facilitate the introduction of dengue into areas historically considered non-endemic, reinforcing the need for internationally coordinated surveillance strategies and adaptable preparedness systems [68,69,70,71,90,91].

Evidence on surveillance and preparedness is stronger in terms of conceptual consistency than operational comparability. Studies frequently differ in data sources, reporting systems, outcome definitions, forecasting horizons, and criteria used to evaluate timeliness or effectiveness [71,72,73,74,75,76,77,78,79,80,81,82,83,84,85,86,87,88,89,90,91]. Early warning systems may perform well in retrospective validation but fail to demonstrate external validity or operational impact when transferred to other settings. Preparedness should therefore be evaluated not only through predictive accuracy, but also through timeliness, usability, institutional uptake, and capacity to trigger appropriate action. Thus, the broad value of integrated surveillance is supported by convergent evidence, whereas the comparative effectiveness of specific platforms and their impact on outbreak outcomes remain less certain and are frequently based on retrospective or context-specific evaluations.

Overall, the literature strongly supports the interpretation of dengue preparedness as a multidimensional public health challenge extending beyond outbreak response alone. Effective prevention and control appear dependent on the capacity of healthcare systems to integrate surveillance, environmental monitoring, preparedness planning, communication strategies, and adaptive governance within rapidly evolving epidemiological scenarios.

### 3.4. Digital Health, Artificial Intelligence, and Mathematical Modelling

Compared with the extensive literature on climate change and surveillance, the evidence concerning digital health and AI in dengue prevention and control appeared considerably more limited and heterogeneous. Nevertheless, the selected studies consistently suggest that digital technologies are progressively reshaping several dimensions of outbreak monitoring, epidemiological forecasting, and public health communication [10,11,12,13,92,93,94,95].

One of the most recurrent themes concerns the growing role of digital epidemiology and real-time data integration in supporting surveillance activities. The increasing availability of environmental, climatic, mobility-related, and epidemiological datasets has facilitated the development of predictive frameworks capable of improving outbreak monitoring and anticipatory preparedness strategies [10,11,12,92,93,94,95,96]. Digital surveillance systems integrating geospatial analysis, climate forecasting, and online data streams may support earlier identification of transmission signals and potentially improve the timeliness of public health responses. Future AI-supported dengue forecasting should be evaluated not only by predictive performance, but also by calibration, external validity, interpretability, timeliness, fairness, and demonstrated usefulness for operational decision-making.

Internal validation alone is insufficient to establish public health utility. Models should be tested prospectively and externally across different climatic, epidemiological, demographic, and surveillance contexts. Reporting should include calibration, sensitivity to missing or delayed data, uncertainty estimates, fairness across populations and territories, and the consequences of false-positive and false-negative alerts. Explainability is particularly important when model outputs influence resource allocation, vector-control interventions, or risk communication. Implementation also requires interoperable data systems, defined institutional responsibilities, trained personnel, and decision thresholds that translate predictions into timely action [92,93,94,95,96,97,98,99].

Several retrieved studies also explored the application of machine learning and AI-based models to dengue prediction. Although methodological heterogeneity remains substantial, many authors describe promising results in forecasting outbreak trends, vector dynamics, and climate-sensitive transmission patterns [92,93,97,98,99]. The literature also highlights limitations related to data quality, model generalizability, interoperability, and overreliance on predictive tools. Digital innovation should therefore complement, not replace, traditional surveillance, as its effectiveness depends on strong reporting systems, laboratory capacity, and public health governance [11,92,94,97]. In this sense, the effectiveness of digital health tools appears closely linked to the broader resilience and adaptability of healthcare systems.

Another emerging aspect concerns the increasing relevance of digital communication ecosystems during infectious disease outbreaks. Social media platforms, online information flows, and rapidly evolving communication environments may significantly influence risk perception, public trust, behavioural responses, and the dissemination of misinformation [13,95,100]. These dynamics appear particularly relevant in rapidly evolving outbreak scenarios, where communication quality may directly affect adherence to prevention measures and engagement with health authorities.

The evidence base remains dominated by retrospective modelling studies and proof-of-concept applications [92,93,94,95,96,97,98,99]. External validation across countries and epidemiological settings is uncommon, while prospective evaluation of effects on public health decisions, resource allocation, or outbreak outcomes remains limited. Comparisons across studies are further complicated by differences in datasets, outcome definitions, model architectures, and reporting standards. Claims of improved preparedness should therefore be reserved for tools that demonstrate reproducibility, interpretability, transportability, and operational usefulness beyond the development dataset. Consequently, evidence that digital tools improve forecasting performance should be distinguished from evidence that they improve real-world preparedness, decision-making, or health outcomes, which remains comparatively limited.

Overall, digital health and AI represent rapidly evolving but still relatively fragmented areas within dengue prevention and control research. Although predictive technologies and digital epidemiology approaches may substantially strengthen surveillance and preparedness capacity, their long-term public health impact will likely depend on integration with robust healthcare systems, effective communication strategies, and interdisciplinary governance frameworks.

### 3.5. Health Communication and Community Engagement

Health communication is a smaller but important research area, showing that public perception, media dynamics, and misinformation can significantly affect dengue outbreak management and prevention [101,102,103,104,105].

One of the most recurrent themes concerns the growing interaction between infectious disease epidemiology and digital communication environments. Social media platforms, online news dissemination, and rapidly evolving information ecosystems contribute to shaping public awareness, risk perception, institutional trust, and behavioural responses during outbreaks [101,106,107,108,109]. In this context, communication is no longer interpreted as a secondary component of outbreak management, but rather as an integral dimension of preparedness and public health governance.

Several studies show that misinformation, distrust, sensationalist media, and inconsistent risk communication can weaken outbreak response by reducing prevention adherence, community engagement, and timely healthcare-seeking [101,104,105,110]. These dynamics appear particularly relevant in rapidly evolving epidemic scenarios characterised by uncertainty, information overload, and high public attention.

A related challenge is the difficulty of sustaining community participation outside periods of acute outbreak visibility. Household source reduction, including the regular emptying, covering, cleaning, or removal of water-holding containers, requires repeated action over time. Participation often declines when perceived risk decreases, when responsibilities are framed as exclusively individual, or when public campaigns are intermittent and unsupported by municipal services. Effective communication should therefore move beyond short-term awareness campaigns and support continuous community engagement, locally feasible prevention practices, feedback mechanisms, and shared responsibility between households and public institutions. Misinformation may further weaken continuity by reducing trust in vector-control recommendations or creating confusion regarding the effectiveness and safety of preventive measures [111,112].

Another important aspect concerns the increasing use of digital communication data as complementary epidemiological indicators. Some studies suggest that online search trends, social media activity, and digital engagement patterns may contribute to identifying changes in public concern, outbreak awareness, and population responses in near real-time [113,114,115]. Although these approaches cannot replace traditional surveillance systems, they may provide useful supplementary information for public health monitoring and communication planning.

The observed literature also points to the need for integrated communication strategies that combine epidemiological accuracy, public trust, cultural adaptation, and digital responsiveness. In the context of dengue outbreaks, effective communication has become increasingly dependent on interdisciplinary collaboration among healthcare professionals, epidemiologists, communication experts, digital platforms, and public institutions [102,103,108,116,117,118].

The evidence in this field is still fragmented and frequently based on cross-sectional surveys, social-media analyses, or context-specific behavioural studies [101,102,103,104,105,106,107,108,109,110,111,112,113,114,115,116,117,118]. Measures of trust, misinformation exposure, risk perception, and adherence are not standardised, and causal relationships are often difficult to establish. More longitudinal and intervention-based research is needed to determine which communication strategies sustain preventive behaviour and community participation over time.

Overall, health communication represents a rapidly evolving but still underexplored component of dengue preparedness and prevention. Beyond the simple dissemination of information, communication emerges as a strategic determinant capable of influencing risk perception, institutional trust, public engagement, and the societal resilience of healthcare systems facing emerging infectious disease threats.

### 3.6. Strengths and Limitations

The principal strength of this review lies in its integration of environmental, urban, epidemiological, technological, communicative, and healthcare-system dimensions within a single preparedness-oriented framework. The structured thematic search strategy and independent screening process also increased the transparency of study selection across heterogeneous domains. Nevertheless, this review has limitations. First, as it was designed as a structured narrative review, the literature selection was interpretative rather than exhaustive. Second, no formal risk-of-bias or quality appraisal was performed. Third, the thematic approach may have favoured broad conceptual integration over detailed evaluation of individual interventions. Finally, although the study-selection process was reported transparently, the review was not designed to provide a comprehensive systematic synthesis of all available evidence. These limitations should be considered when interpreting the conclusions.

## 4. Conclusions

Dengue preparedness requires a transition from reactive outbreak control toward anticipatory, integrated, and locally adaptable public health action. Surveillance systems should combine epidemiological, entomological, climatic, environmental, and mobility-related information and should be evaluated according to their capacity to trigger timely decisions. Vaccination should be integrated with serotype monitoring, safety surveillance, transparent communication, and sustained vector control. Urban prevention should prioritise reliable water infrastructure, waste management, household source reduction, microclimatic risk assessment, and targeted interventions in socially and environmentally vulnerable areas.

Digital epidemiology, mathematical modelling, and artificial intelligence may strengthen early warning and resource allocation, but only when models are externally validated, interpretable, interoperable, and connected to clearly defined operational responses. Risk communication should be treated as a continuous preparedness function rather than an activity activated only during outbreaks. Long-term community participation requires sustained engagement, institutional trust, municipal support, and shared responsibility.

Future research should prioritise prospective evaluation of early warning systems, comparative assessment of vaccination strategies, validation of AI-supported models across heterogeneous settings, fine-scale analysis of urban and climatic risk, and intervention studies on communication and community engagement. Dengue preparedness should ultimately be assessed by whether public health and healthcare systems can detect changing risk early, protect vulnerable populations, translate evidence into action, and maintain trust before transmission escalates.

## Figures and Tables

**Table 1:** Database search outputs and records selected for narrative synthesis across the predefined thematic domains.

Thematic Area	Scopus	Web of Science	PubMed	Selected for Synthesis
Climate change and environmental transformation	2366	1881	1064	23
Urbanisation and urban health	1063	1780	679	24
Surveillance, vaccination, and integrated preparedness	6758	5569	4736	18
Digital health, artificial intelligence, and mathematical modelling	406	300	176	9
Health communication and community engagement	315	505	124	16

## Data Availability

No new data were generated or analysed in this study. All sources discussed in this review are cited in the References list.

## Supplementary Materials

The following supporting information can be downloaded at: https://www.mdpi.com/article/10.3390/medicina62071333/s1, Table S1. Main thematic search strategies adopted for the narrative review.
