# Supplementary material for: Rethinking Dengue Preparedness in the Era of Climate Change, Urbanisation, and Digital Health: A Structured Narrative Review
Source: Medicina (Kaunas). 2026 Jul 10;62(7):1333. doi: 10.3390/medicina62071333 (PMC13413979; doi:10.3390/medicina62071333)
Supplement: Supplementary file 1 [file medicina-62-01333-s001.zip › Supplementary_Table_S1_Dengue_final_clean.pdf]

Supplementary Table S1. Main thematic search strategies adopted for the narrative review.

Thematic area	Search string
Climate change and environmental transformation	(“dengue”) AND (“climate change” OR “global warming”)
Urbanization and urban health	(“dengue”) AND (“urbanization” OR “urban health” OR “built environment” OR “urban sprawl”)
Surveillance, vaccination, and integrated preparedness	(“dengue”) AND (“preparedness” OR “public health preparedness” OR “surveillance” OR “early warning systems”)
Digital health, artificial intelligence, and mathematical modelling	(“dengue”) AND (“digital health” OR “digital epidemiology” OR “artificial intelligence” OR “digital surveillance”)
Health communication and community engagement	(“dengue”) AND (“misinformation” OR “risk communication” OR “health communication” OR “social media”)
